# Establishment of a simple and efficient *Agrobacterium*-mediated transformation system for *Phytophthora palmivora*

**DOI:** 10.1186/s12866-016-0825-1

**Published:** 2016-09-06

**Authors:** Dongliang Wu, Natasha Navet, Yingchao Liu, Janice Uchida, Miaoying Tian

**Affiliations:** 1 Department of Plant and Environmental Protection Sciences, University of Hawaii at Manoa, 3190 Maile Way, St. John 317, Honolulu, HI 96822 USA; 2 Present Address: College of Plant Protection, Agricultural University of Hebei, Baoding, China

**Keywords:** *Agrobacterium*-mediated transformation, *Phytophthora palmivora*, Oomycete, GFP, Copy number

## Abstract

**Background:**

As an agriculturally important oomycete genus, *Phytophthora* contains a large number of destructive plant pathogens that severely threaten agricultural production and natural ecosystems. Among them is the broad host range pathogen *P. palmivora*, which infects many economically important plant species. An essential way to dissect their pathogenesis mechanisms is genetic modification of candidate genes, which requires effective transformation systems. Four methods were developed for transformation of *Phytophthora* spp., including PEG(polyethylene glycol)/CaCl_2_ mediated protoplast transformation, electroporation of zoospores, microprojectile bombardment and *Agrobacterium-*mediated transformation (AMT). Among them, AMT has many advantages over the other methods such as easy handling and mainly generating single-copy integration in the genome. An AMT method previously reported for *P. infestans* and *P. palmivora* has barely been used in oomycete research due to low success and low reproducibility.

**Results:**

In this study, we report a simple and efficient AMT system for *P. palmivora*. Using this system, we were able to reproducibly generate over 40 transformants using zoospores collected from culture grown in a single 100 mm-diameter petri dish. The generated GFP transformants constitutively expressed GFP readily detectable using a fluorescence microscope. All of the transformants tested using Southern blot analysis contained a single-copy T-DNA insertion.

**Conclusions:**

This system is highly effective and reproducible for transformation of *P. palmivora* and expected to be adaptable for transformation of additional *Phytophthora* spp. and other oomycetes. Its establishment will greatly accelerate their functional genomic studies.

## Background

Oomycetes, belonging to phylum Oomycota within the kingdom Straminipila, form a diverse group of fungus-like eukaryotes that include many destructive pathogens of plants and animals [1–3]. Among them, the genus *Phytophthora* contains over 100 species with a large number of them as devastating plant pathogens that severely threaten agricultural production and natural ecosystems [4–6], such as the notorious potato famine pathogen *P. infestans* which causes late blight of tomato and potato [7], soybean root and stem rot pathogen *P. sojae* [8], sudden oak death pathogen *P. ramorum* that is endangering oak trees along the Pacific coast of US [9], the vegetable blight pathogen *P. capsici* which attacks various vegetable crops [10], and the wide-host-range *P. cinnamomi and P. palmivora* [6, 11]. *P. palmivora* infects numerous plant species, including many economically important hosts such as papaya, cacao, rubber tree, citrus, coconut and black pepper [6].

With the availability of the genome sequences of several *Phytophthora* spp. and *in silico* identification of hundreds of effectors in each genome [12–15], one of the major tasks is to link gene sequences to their biological functions using genetic approaches, which requires highly effective transformation systems. Four methods were developed for transformation of *Phytophthora* spp., including PEG(polyethylene glycol)/CaCl_2_ mediated protoplast transformation, electroporation of zoospores, microprojectile bombardment and *Agrobacterium*-mediated transformation (AMT). The PEG/CaCl_2_ protoplast transformation method was first established by Judelson et al. [16] to transform *P. infestans* and now it has been commonly used in transformation of several *Phytophthora* pathogens including *P. infestans*, *P. parasitica* [17], *P. sojae* [18–20], *P. palmivora* [21], *P. cactorum* [22], and *P. capsici* [23]. This method is labor intensive and requires large amounts of starting materials. Moreover, it often encounters difficulties in generating protoplasts and low rate of regeneration of protoplasts [24]. Microprojectile bombardment bypasses the need of protoplasting, and has been used for transformation of *P. infestans* [25, 26]. However, as it requires specialized equipment, thus far its application has been very limited. Electroporation of zoospores is gaining popularity due to its ease to be performed and it has been used for transformation of *P. capsici* [27], *P. infestans* [26, 28, 29] and *P. palmivora* (Gumtow and Tian, unpublished data). A common disadvantage associated with the above three transformation approaches is that they often generate multi-copy integration in the genome. Although a higher copy number may be associated with higher level of gene expression and gene silencing [26], multi-copy integration randomly disrupts multiple genes varying among transformants and therefore complicates gene function analyses. As a result, only very limited number of *Phytophthora* genes has been functionally characterized through genetic modification. In contrast, AMT circumvents this issue as it usually generates the integration of one or two copies [30]. In addition, it does not require protoplasting and specialized equipment such as gene gun (biolistic particle delivery system) or electroporator, and is easy in handling. While the other methods requires large amount of plasmid DNA (20 to 30 μg for PEG/CaCl_2_ mediated protoplast transformation and electroporation, 1 μg for bombardment) to get a decent number of transformants [16, 25, 27], AMT doesn’t need DNA preparation once the plasmids are transformed into *Agrobacteria*. An AMT method was previously reported for *P. infestans* and *P. palmivora* [30]*,* however, its further use in oomycete research has never been reported likely due to low success and low reproducibility of generating transformants.

In the present study, we largely modified the AMT method developed by Vijn and Govers [30] and established a simple, efficient and highly reproducible system to transform *P. palmivora.* This approach will greatly facilitate dissection of *P. palmivora* pathogenesis mechanisms by functional genomic studies, and is expected to be adaptable for transforming other oomycetes.

## Methods

### *Phytophthora palmivora* strain and culture conditions

*Phytophthora palmivora* strain P1, isolated from an infected papaya plant grown in Poamoho research station, University of Hawaii at Manoa, was used throughout this study and routinely cultured on 10 % unclarified V8 agar under 12 h light/12 h dark at room temperature (around 22 °C).

### Construction of plasmids

**Fig. 1:**
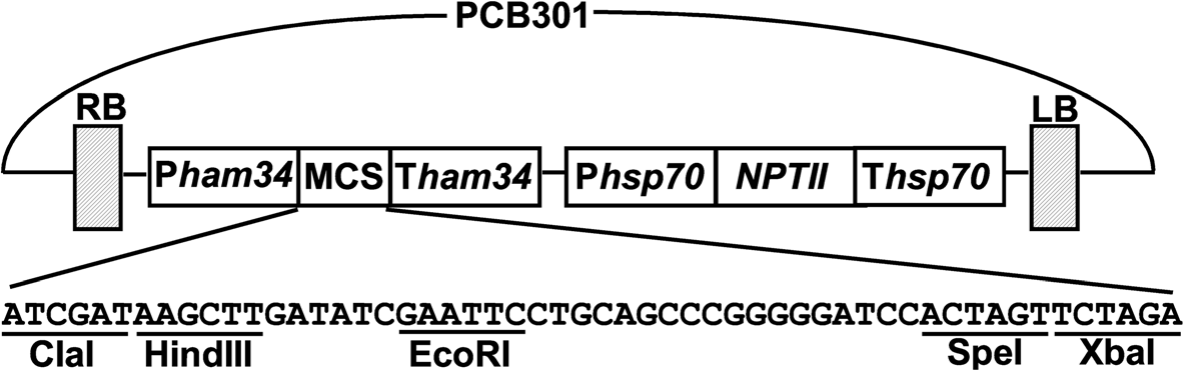
Schematic representation of pCB301TOR. RB, T-DNA right border; LB, T-DNA left border. P*ham34* and P*hsp70*, promoters of *ham34* and *hsp70*. T*ham34* and T*hsp70*, terminators of *ham34* and *hsp70*. MCS, multiple cloning site. *NPTII*, neomycin phosphotransferase II

To generate a binary vector that can be used to transform *P. palmivora* via *Agrobacterium tumefaciens*-mediated transformation, the mini binary vector pCB302 previously developed for plant transformation [31] was utilized. Briefly, the *bar* gene expression cassette was removed with restriction enzymes KpnI and SacI (described as SstI in Xiang et al., 1999), and replaced with the fragment from 1011 bp to 4018 bp of the oomycete expression vector pTOR (GenBank: EU257520.1) [32]. This fragment contains two gene expression cassettes. The first cassette contains *Bremia lactucae* Ham34 promoter (1011–1575 bp), a multiple cloning site (1576–1688 bp) to clone genes to be transformed, and Ham34 terminator (1689–2209 bp). The second cassette contains *Bremia lactucae* Hsp70 promoter, *NPTII* gene used to select transformants resistant to G418, and Hsp70 terminator. The primers PHam34-FSacI (5’- gcggagctcTCTGATGGACAAAGGGTCGCCT-3’) and THsp70-RKpnI (5’-gcgggtaccAAGCACAATAGGCCCAGACTC-3’) were used to amplify this fragment using pTOR as template. The template-specific sequence is shown in uppercase, and the introduced SacI and KpnI restriction sites are underlined. The generated plasmid was designated as pCB301TOR (Fig. 1). The plasmid pCB301TOR-GFP was generated by cloning PCR-amplified DNA fragment corresponding to the GFP protein-encoding sequence into EcoRI and SpeI sites of pCB301TOR. Primers GFP-FEcoRI (5’-gcggaattcATGGTGAGCAAGGGCGAG-3’) and GFP-RSpeI (5’-gcgactagtTTACTTGTACAGCTCGTCCATGC-3’) were used to amplify the GFP fragment from pIGPAPA [33]. The gene-specific sequence is shown in uppercase, and the introduced restriction sites are underlined.

### Preparation of *P. palmivora* zoospores for transformation

Seven-day-old *P. palmivora* culture grown on 10 % unclarified V8 agar in a petri dish was flooded with 10 ml ice-cold sterile miliQ water and incubated at room temperature for 30 min to release the motile zoospores. The zoospore suspension was gently recovered without touching mycelia using a pipette. The concentrations of the zoospores were usually 2–5 × 10^6^/ml.

### Preparation of *Agrobacterium tumefaciens* for transformation

*Agrobacterium tumefaciens* EHA105 strains containing pCB301TOR or pCB301TOR-GFP, which were stored at −80 °C freezer for long-term storage, were streaked on a LB agar plate supplemented with 15 μg/ml of rifampicin and 50 μg/ml of kanamycin and grew at 28 °C for 2 days. The culture can be stored at 4 °C up to one week until the day before transformation. Then the culture was spread on a new LB agar plate with antibiotics and grew overnight. Before transformation, the bacterial cells were scraped from the LB plate, suspended and further diluted to various concentrations in *Agrobacterium* induction medium (IM)*,* which consisted of liquid minimal medium (MM) [34] supplemented with 200 μM acetosyringone in 50 ml sterile conical centrifuge tubes. The centrifuge tube with *Agrobacterium* suspension was wrapped in foil and incubated at room temperature for 2 h on a platform shaker with gentle agitation to induce *vir* gene expression.

### *Agrobacterium tumefaciens*-mediated transformation of *P. palmivora* zoospores

Equal volumes of *Agrobacterium* suspension and zoospore suspension prepared as above were mixed gently by swirling the tubes and incubated at room temperature in dark for 2 h. Every 330 μl of the mixture was evenly spread onto a 5 × 5 cm piece of sterilized Hybond N^+^ membrane (GE Healthcare) placed atop of solid IM containing 200 μM acetosyringone and 1.5 % agar and dried in the hood for about 10 min. The IM agar plates were kept in dark at room temperature for 2 days. After that the Hybond N^+^ membranes were transferred upside down to Plich medium [35] agar plates supplemented with 30 μg/ml G418 and 200 μM cefotaxime (the side with *Agrobacteria* and zoospores facing the medium). Any air bubble between the membrane and medium was squeezed out with a pair of forceps to make sure that the membrane was in good contact with the medium. The plates were incubated at room temperature under 12 h light/12 h dark condition. After three days, the membranes were removed and the plates were kept under the same condition to allow the G418-resistant colonies to grow. The transformants usually appeared 1–3 days after the membranes were removed. The G418-resistant colonies were transferred to 10 % V8 agar plates with 30 μg/ml G418 and 200 μM cefotaxime. One week later, single zoospores from each transformant were isolated as described previously [36] and grown on 10 % V8 agar plates with 30 μg/ml G418 to obtain single zoospore-derived transformants.

### Visualization of GFP

Mycelia and sporangia of the transformants grown on 10 % V8 agar media were used to visualize the expression of GFP using a Zeiss Axio Scope.A1 fluorescence microscope. The wild type *P. palmivora* strain P1 was used as a negative control.

### DNA isolation and Southern blot

For isolation of DNA used for Southern blot, agar plugs of *P. palmivora* transformants were inoculated in 100 × 15 mm petri-dishes containing 20 ml of liquid Plich medium [35]. The cultures were incubated at room temperature in dark for 7 days. The mycelia were harvested by filtering through Whatman filter paper under a vacuum and flash frozen in liquid nitrogen. The frozen mycelia were ground into fine powder in liquid nitrogen with a mortar and pestle. Genomic DNA was extracted from the mycelia using a standard phenol and chloroform protocol [37]. Briefly, the ground mycelia were lysed in Isolation buffer (150 mM EDTA, 50 mM Tris-HCl pH 8.0, 1 % Sarkosyl, 300 mg/l Proteinase K) and extracted once with Tris-saturated phenol, twice with phenol:chloroform:isoamyl alcohol (25:24:1), once with chloroform:isoamyl alcohol (24:1). Then DNA was precipitated with ethanol and the contaminating RNA removed by RNase A treatment. Another round of phenol:chloroform:isoamyl alcohol and chloroform:isoamyl alcohol extractions were performed to further purify the RNase-treated DNA followed by ethanol precipitation. The final DNA pellets were resuspended in 1 × TE buffer (10 mM Tris-HCl, 1 mM EDTA, pH 8.0).

For Southern blot analysis to determine the copy number of T-DNA insertions in *P. palmivora* transformants, 15 μg of genomic DNA was digested with EcoRI and run on 0.8 % agarose gel in 1 × TBE buffer (220 mM Tris; 180 mM Borate; 5 mM EDTA; pH 8.3). The separated DNA was then transferred and fixed onto Amersham Hybond-N+ membrane (GE Healthcare) using capillary blotting per manufacturer’s manual. The probe used was a DNA fragment encoding the open reading frame (ORF) of NPTII amplified using Phusion DNA polymerase (Thermo Scientific) with the plasmid pCB301TOR-GFP as template. The PCR product was purified using QiaQuick PCR Purification Kit (Qiagen) and labeled with biotin using Biotin DecaLabel DNA Labeling Kit (Thermo Scientific). After labeling, the probe was purified with QiaQuick PCR Purification Kit again. Hybridization and detection were performed using Biotin Chromogenic Detection Kit (Thermo Scientific) following the manufacturer’s instructions.

## Results

### Construction of a binary vector for *Agrobacterium*-mediated transformation of oomycetes

We constructed a binary vector pCB301TOR (Fig. 1) for *Agrobacterium*-mediated transformation (AMT) of *P. palmivora* by utilizing the backbone of a mini binary vector series developed for plant transformation [31]. The backbone sequence of pCB301TOR including T-DNA left border (LB) and right border (RB) is the same as pCB301 (GenBank: AF139061.1) [31]. Within the LB and RB, there are two gene expression cassettes derived from the oomycete expression vector pTOR (GenBank: EU257520.1). The Ham34 promoter-multiple cloning site-Ham34 terminator cassette is used to clone the gene to be transformed; the hsp70 promoter-*NPTII*-hsp70 terminator is used to express *NPTII* gene for selecting transformants on G418-containing media. The length of pCB301TOR is 6485 bp. The unique restriction sites can be used for cloning are ClaI, HindIII, EcoRI, SpeI and XbaI in the order from Ham34 promoter to Ham34 terminator (Fig. 1). The selection maker in *E. coli* and *Agrobacterium tumefaciens* is Kanamycin. Using this vector, *P. palmivora* was successfully transformed with GFP via AMT, suggesting that pCB301TOR is effective in transforming *P. palmivora.* As this vector utilizes Ham34 and Hsp70 promoters and terminators, which have been widely used in transformation of various oomycetes [16, 17, 19, 27, 38, 39], it is expected to be suitable for transforming other oomycetes in addition to *P. palmivora.*

### Establishment of a simple and efficient protocol for *Agrobacterium tumefaciens*-mediated transformation of *P. palmivora*

**Fig. 2:**
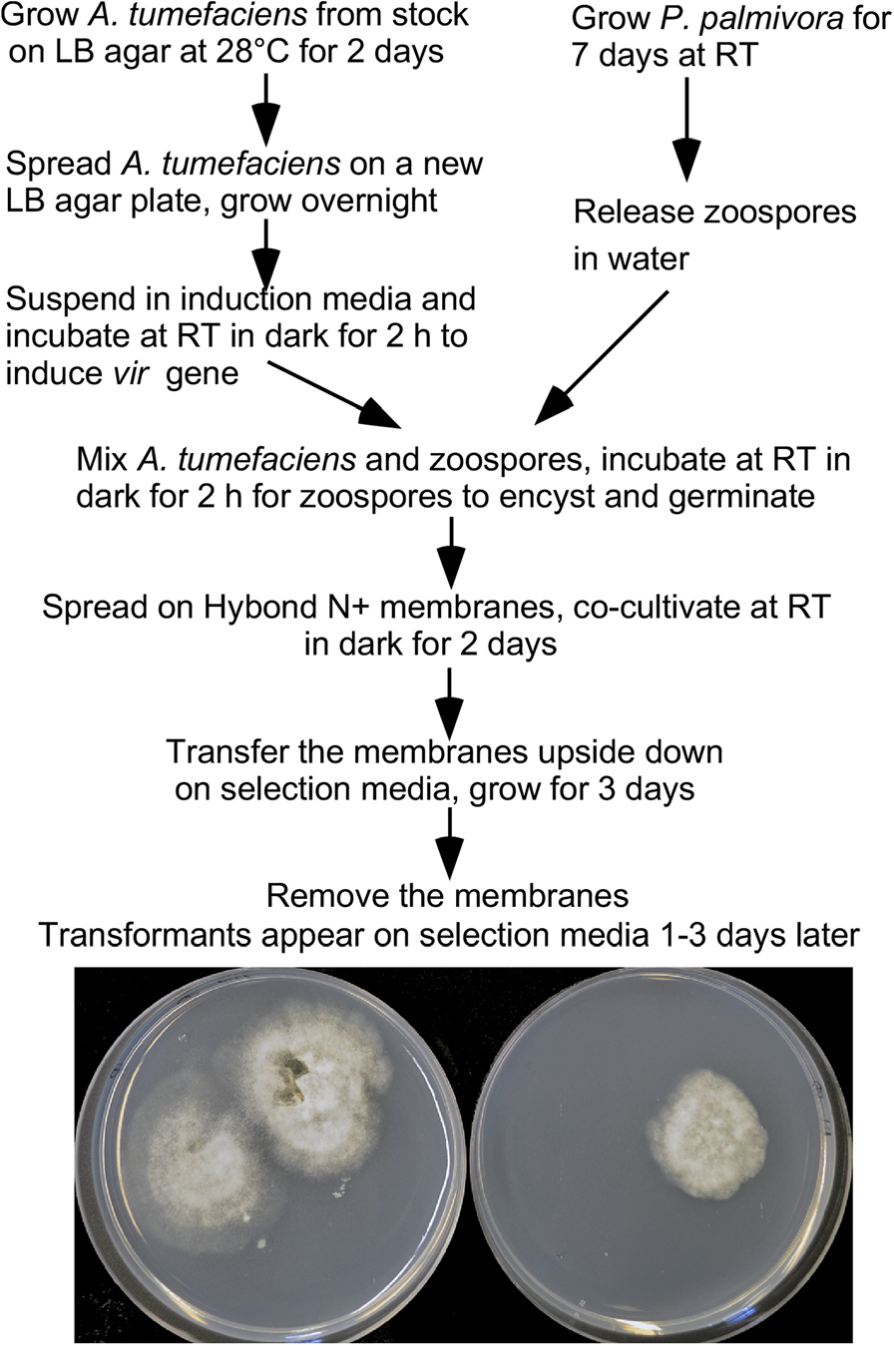
An outline of the method developed for *Agrobacterium*-mediated transformation of *P. palmivora*. Three representative transformants from two selection plates are shown in the picture at the bottom

Using the EHA105 strain containing pCB301TOR, we initially followed the method described by Vijn and Govers [30] to transform *P. palmivora*. Repeated experiments produced no real transformants except several false positives. The G418 resistant colonies obtained failed to sporulate and PCR using both HSP70 promoter primers and NPTII primers yielded no products (data not shown). Consistent with what was suggested by Vijn and Govers [30], their method may still be far from optimal to be used for transforming *P. palmivora*. To this end, we drastically modified the method using the EHA105 strain containing pCB301TOR-GFP to make it simple, efficient and highly reproducible. An outline of the method is illustrated in Fig. 2 and the details are described in Materials and Methods.

We modified the method to prepare *A. tumefaciens* for transformation. Instead of growing *A. tumefaciens* in liquid media, we grew them on the LB agar plates and then resuspended the collected cells in *Agrobacterium* induction medium (IM), which were directly used for co-incubation with zoospore suspension.

To prepare *P. pamivora* zoospore suspension, we covered the sporulating cultures on 10 % V8 agar plate with 10 ml ice-cold water to allow zoospores to be released into the water. This zoospore suspension was directly used for transformation. Compared with the method described by Vijn and Govers [30], this method skipped sporangium collection and purification steps. As *P. palmivora* usually easily produces and releases high amount of zoospores, we were able to routinely get zoospores at concentrations 2–5 × 10^6^/ml without any concentration procedures. This simple method reduced the handling procedures and time, and therefore likely minimized any adverse effect on zoospore survival and germination.

After co-incubating zoospores with *A. tumefaciens* cells for 2 h, the mixtures were directly placed on a square piece of Hybond N+ membrane of 5 × 5 cm, which covered the major area of the petri dish. Any centrifugation step that might cause the loss or damage of the germinated cysts or *A. tumefaciens* cells was avoided.

After co-cultivation of zoospores with *A. tumefaciens* cells on hybond N+ membrane placed on top of IM agar plate containing 200 μM acetosyringone, initially we followed the method by Vijn and Govers [30] by cutting the membranes into 1 cm^2^ pieces and transferred upside down to Plich agar plates supplemented with G418 for selection of the transformants and cefotaxime to kill the *A. tumefaciens*. However, this method took hours of time to cut the membranes and sterilize scissors and forceps between membranes. In addition, redundant transformants might be recovered from multiple small membrane pieces originated from the same big piece either because they were next to each other or due to the cross-contamination resulted from repeated use of the scissors and forceps without sterilizing them. To save time and avoid producing redundant transformants, we transferred the whole 5 × 5 cm piece of membrane upside down onto the selection media and removed the membrane three days later to allow the transformants to appear on the media (Fig. 2).

To determine the optimal concentration of *A. tumefaciens* used for *P. palmivora* transformation, varying concentrations of *A. tumefaciens* expressing pCB301TOR-GFP with OD_600_ of 0.1, 0.2, 0.4 and 0.8 were mixed with equal volume of zoospore suspension and tested for transformation efficiency. G418-resistant GFP-expressing transformants were obtained with all concentrations, with OD_600_ at 0.4 produced the highest number of transformants (Table 1). Increasing OD_600_ above 0.4 reduced numbers of transformants likely due to overgrowth of *Agrobacteria* inhibited growth of *P. palmivora*. When the *Agrobacterium* concentration at OD_600_ = 0.4 was used for transformation, an average of 27 G418-resistant transformants per 10^7^ zoospores were obtained from three independent transformation experiments (Table 1). As we were able to routinely get 8 ml of zoospores at concentrations 2–5 × 10^6^/ml from *P. palmivora* culture grown in a 100 mm-diameter petri dish, a range of 43–128 transformants could be generated when using culture from a single 100 mm-diameter petri dish for the transformation experiment.

**Table 1:** Transformation of *P. palmivora* using various concentrations of *A. tumefaciens* EHA105 expressing pCB301TOR-GFP

OD_600_ of *Agrobacteria*	Data from 1 representative experiment					Data from 3 experiments
	Zoospore concentration (/ml)	Zoospore volume (ml)	Number of G418-resistant transformants	Number of transformants with detectable GFP signal	Number of G418-resistant transformants/10^7^ zoospores	Average number of G418-resistant transformants/10^7^ zoospores
0	3.3 × 10^6^	2.5	0	0	0	0
0.1	3.3 × 10^6^	2.5	13	10	16	22 ± 5
0.2	3.3 × 10^6^	2.5	16	15	19	21 ± 2
0.4	3.3 × 10^6^	2.5	25	23	30	27 ± 5
0.8	3.3 × 10^6^	2.5	13	13	16	18 ± 4

**Fig. 3:**
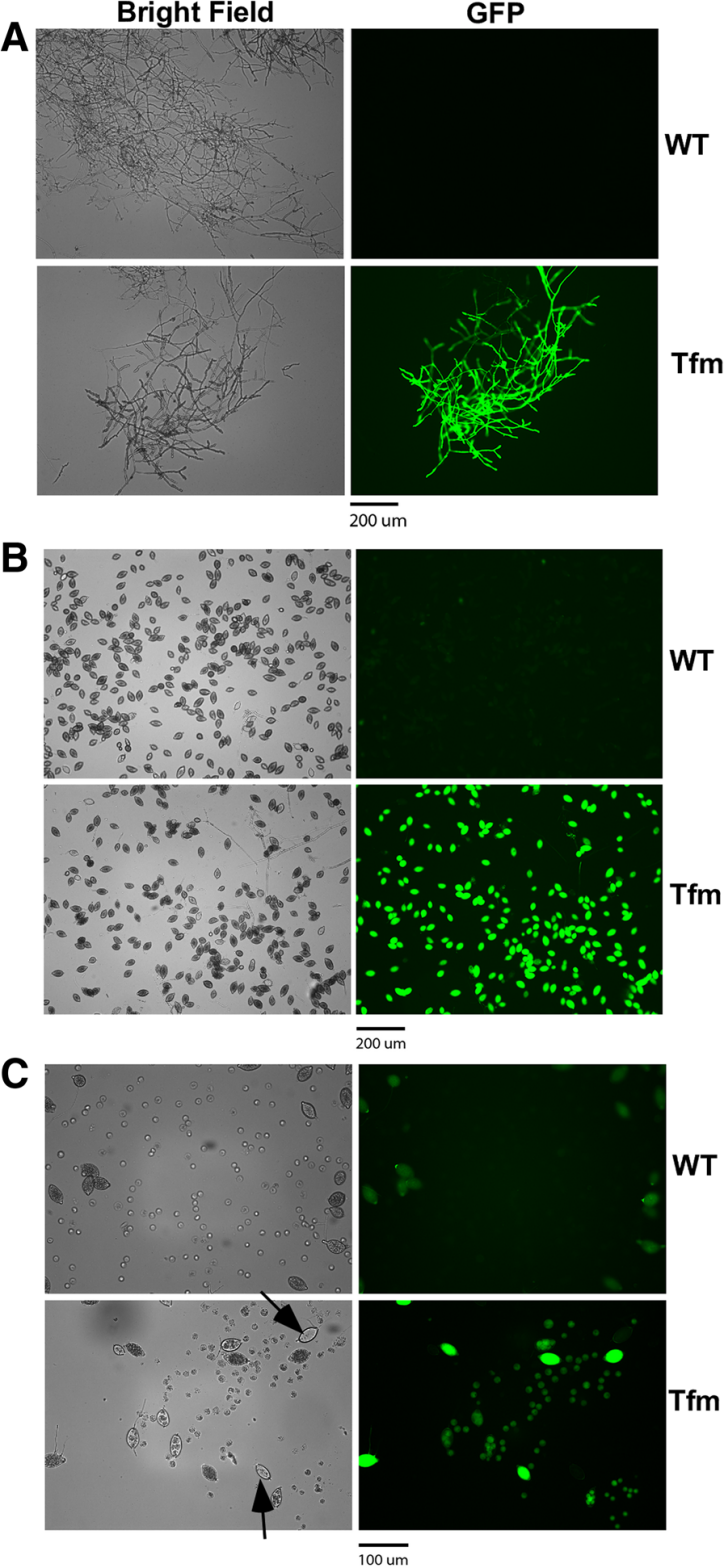
Expression of GFP in a representative transformant transformed with pCB301TOR-GFP. Photographs of mycelia (**a**), sporangia (**b**), sporangia and immobile zoospores (**c**) of the wild type strain (WT) and a representative GFP transformant (Tfm) under bright field and GFP fluorescence channel (GFP). Note that empty sporangia (indicated by arrows) did not show GFP fluorescence; Sporangia of the wild type strain showed some auto-fluorescence under GFP channel when 20× objective was used. Scale bars are shown at the bottom of each panel

Few false positives were produced. 16 G418-resistant *P. palmivora* clones were randomly selected and tested for the presence of transgenes using primers based on Hsp70 promoter sequence. All transformants tested were shown to be real transformants (data not shown). A high percentage of G418-resistant transformants expressed GFP (Table 1, Fig. 3). The GFP signals were detected in mycelia (Fig. 3a), zoospore-containing sporangia (Fig. 3b), and zoospores (Fig. 3c).

The protocol we developed was shown to be efficient and highly reproducible. Using this method, we also transformed *P. palmivora* with two *P. palmivora* genes under characterization, including a cystatin-like extracellular protease inhibitor and a putative effector with an RxLR (Arg-x-Leu-Arg) translocation motif [40]. Both genes were cloned to pCB301TOR and transferred to *A. tumefaciens* EHA105 and AGL1, respectively. Over 40 transformants were obtained for each construct using zoospores collected from culture grown on 10 % V8 agar in a single 100 mm-diameter petri dish.

### T-DNA copy number of *P. palmivora* transformants

**Fig. 4:**
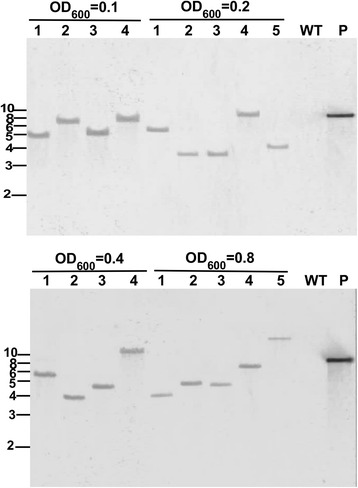
Southern blot analyses of *P. palmivora* transformants. Total DNA was digested using EcoRI and the blots were probed with a biotin-labeled DNA fragment encoding the open reading frame of NPTII. WT, wild-type *P. palmivora* strain. P, EcoRI-digested plasmid pCB301TOR-GFP. The numbers on the left of the blots represent the sizes (Kb) of DNA bands from NEB 1Kb DNA ladder

To determine the copy number of T-DNA insertions in *P. palmivora* transformants generated using different concentrations of *A. tumefaciens* EHA105 containing pCB301TOR-GFP. We selected 5 transformants for each concentration to isolate DNA for Southern blot analyses. Due to low DNA yield of 1 transformant at OD_600_ = 0.1 and 1 transformant at OD_600_ = 0.4, we did not include these two transformants for further analysis. For the remaining 18 transformants, the Southern blot analyses were performed with NPTII as the probe and genomic DNA digested with EcoRI. As expected, no hybridization signal was observed for the wild-type strain (Fig. 4). For all 18 transformants, a single hybridized band appeared suggesting that all transformants tested contained a single copy of T-DNA insertion (Fig. 4).

## Discussion

In the present study, we established a simple and efficient system to transform *P. palmivora* using *Agrobacterium*-mediated transformation. We constructed a binary vector pCB301TOR (Fig. 1) by utilizing the backbone of a mini binary vector series developed for plant transformation [31], and two gene expression cassettes derived from the oomycete expression vector pTOR (GenBank: EU257520.1). Using this binary vector and the protocol we developed, we have reproducibly obtained transformants expressing GFP. All 18 transformants tested for copy numbers were shown to contain a single copy of T-DNA integration. We have also successfully generated transformants ectopically expressing a cystatin-like extracellular protease inhibitor and an RxLR effector of *P. palmivora.* Using *Agrobacteria* at a concentration of OD_600_ = 0.4 and zoospores harvested from a single culture plate in a 100 mm-diameter petri dish, we could obtain 40 to over 100 transformants. This system is highly reproducible. With this system, we haven’t failed in getting transformants in any *P. palmivora* transformation experiment we have performed so far. The establishment of this system will be instrumental in dissecting the pathogenesis mechanism of *P. palmivora* using genetic approaches.

The AMT system described in this study was established based on the method developed by Vijn and Govers [30] with drastic modifications. There are several major differences between the protocol by Vijn and Govers [30] and ours that likely determine whether the transformation would be successful. In Vijn and Gover’s method, *Agrobacteria* were grown in induction medium containing 100 μM acetosyringone for 5 h to induce *vir* gene expression, followed by washing twice with sterile water before mixing with zoospores for co-incubation. As acetosyringone was washed away, it was absent during co-incubation of zoospores and *Agrobacteria*. In contrast, in our modified method, *Agrobacteria* grown on solid LB medium was resuspended in liquid induction medium containing 200 μM acetosyringone to induce *vir* gene expression for 2 h, which was then directly used for co-incubation with zoospores. Acetosyringone was present during the whole co-cultivation process. Moreover, higher concentration of acetosyringone was used in our method than Vijn and Govers’s method. The presence of higher concentrations of acetosyringone during the whole co-cultivation process may have contributed to the effectiveness of our method. In addition, Vijn and Govers [30] mixed large volume (50 ml) of zoospores (10^6^ zoospores/ml) with a small volume (1 ml) of *Agrobacteria* (OD600 = 0.25) for transformation, which likely diluted the *Agrobacteria* too much leading to less chance to infect zoospores and reduced virulence. In our method, we routinely mixed equal volumes of zoospores (2–5 × 10^6^ zoospores/ml) and *Agrobacteria* (OD600 = 0.4), which provided higher chance for *Agrobacteria* to contact and infect zoospores.

There are other modifications that could contribute to the success of the modified method. In Vijn and Govers’s method [30], zoospores were induced to encyst by manual shaking for 2 min after 30 min of co-incubation with *Agrobacteria*. On the contrary, our methods allowed zoospores to encyst and germinate without disturbance. We noticed that the zoospores encysted by themselves during the co-incubation period without the shaking step, which might dislodge the *Agrobacteria* from zoospores or affect the viability of zoospores. In addition, in Vijin and Govers’s method [30], the zoospore-*Agrobacterium* mixture was centrifuged before being placed on the Hybond N+ membrane on the co-cultivation media. The centrifugation step might have detrimental effect to the germinated zoospores. In our protocol, the mixture was directly spread on the membrane.

Other modifications we made were to simplify the protocol as much as possible to save time, minimize adverse effects on zoospores and *Agrobacteria*, and reduce redundant transformants. For the zoospore preparation, instead of collecting and concentrating sporangia followed by zoospore release, we added ice-cold water directly on the culture plate to release zoospores, which were directly used to mix with *Agrobacterium* suspension. For the *Agrobacteria* preparation, we grew and collected the cells on solid medium without the need to wash the antibiotics away after initial growth. After co-cultivation of *Agrobacteria* and *P. palmivora* on induction medium agar plates, we simply place the hybond N+ membrane pieces upside down on top of the selection plates without cutting the membranes into small pieces to save handling time and reduce redundant transformants.

The AMT system we developed for *P. palmivora* is expected to be applicable for other oomycetes with necessary modifications. For species with lower sporangium production, sporangia can be harvested from several plates of culture. After filtering through nylon mesh (40–50 μm), the sporangia can be concentrated by centrifugation followed by releasing of zoospores with a smaller volume. We also used this procedure for *P. palmivora* transformation and the transformation efficiency was similar as when the zoospores were directly released from the culture.

## Conclusions

We developed a simple, efficient and highly reproducible *Agrobacterium*-mediated transformation system for *P. palmivora,* which has the potential to be used to transform other oomycetes. The establishment of this system will serve as an invaluable tool to accelerate functional genomics studies of *P. palmivora* and other oomycetes.

## Abbreviations

AMT: *Agrobacterium-*mediated transformation
GFP: Green fluorescent protein
IM: Induction medium
NPTII: Neomycin phosphotransferase II
PEG: Polyethylene glycol
